# Review of the species of *Michotamia* from China with a description of a new species (Diptera, Asilidae)

**DOI:** 10.3897/zookeys.184.2871

**Published:** 2012-04-21

**Authors:** Lili Zhang, Aubrey Scarbrough, Ding Yang

**Affiliations:** 1 Key Laboratory of Zoological Systematics & Evolution, Institute of Zoology, Chinese Academy of Sciences, Chaoyang, Beijing, 100101, China; 2 Department of Entomology, University of Arizona, Tucson, AZ 85721, USA; 3 Department of Entomology, China Agricultural University, Haidian, Beijing 100193, China

**Keywords:** Diptera, Asilidae, *Michotamia*, new species, China

## Abstract

Three species of *Michotamia* are recorded from China. Of these *Michotamia aurata* (Fabricius, 1794) was previously reported from Hainan and Taiwan. *Michotamia assamensis* Joseph & Parui, 1995 is recorded from China and Laos for the first time, and *Michotamia yunnanensis* **sp. n**., is described and figured. A key to the known species from China is provided. A new name, *Michotamia subnigra*, is given to *Michotamia nigra* Scarbrough & Hill, 2000, which is preoccupied by *Michotamia nigra* (Meijere, 1911).

## Introduction

The species of *Michotamia* Macquart, 1838 are distinguished from the other Ommatiinae genera by the elongated postpedicel (see Stuckenberg 1999), which is at least 1.5 times longer than the combined length of the scape and pedicel. Of the known 24 species, 22 occur in the Oriental Region (Joseph and Parui 1983, 1984, 1987, 1995; Scarbrough and Hill 2000; Tomasovic and Grootaert 2003, 2008). The remaining two are from the Afrotropical and the Australasian/Oceanian regions, respectively (Geller-Grimm 2003/2004).The species from China remain poorly known with only one species, *Michotamia aurata* (Fabricius, 1794), reported from Hainan (Hua 1985) and Taiwan (Joseph and Parui 1998). Here *Michotamia assamensis* Joseph and Parui is recorded from China and Laos for the first time, and a new species, *Michotamia yunnanensis* sp. n., is described and figured. A key to the known species of *Michotamia* from China is included. Types are deposited in Institute of Zoology (IOZ), Chinese Academy of Sciences.

## Taxonomy

### Key to the known species of *Michotamia* (male) from China

**Table:** 

1	Hind femur reddish-yellow or brownish-yellow; apical 1/3 of epandrium abruptly curved dorsally, apex obtuse, about 1/3 as wide as epandrium at middle distance from base to apex in lateral view	*Michotamia aurata*
–	Hind femur largely or at least dorsally black; epandrium wide, margins parallel from base to apex, apex with a weak emargination and prominent dorsal and ventral corners	2
2	Wing hyaline, microtrichia sparse, most abundant apically (Figs 6–7); postpedical 3.5 times combined length of pedicel and scape; fore & mid femora yellow; dorsal surface of hind femur mostly blackish-brown with narrow yellow basally; hind femur narrow, about 7 times longer than diameter medially, blackish dorsally and extending anteriorly, remaining yellowish; apex of epandrium somewhat angular with dorsal corner produced well beyond ventral corner (Figs 2–5)	*Michotamia yunnanensis* sp. n.
–	Wing brownish-yellow, darkest anteriorly, microtrichia wide spread; fore & mid femora yellowish-orange, black extending from base to near apex dorsally & anteriorly; hind femur wider, 5 times longer than diameter medially, mostly black, basal 1/5 reddish-yellow or yellow; apex of epandrium subtruncate, dorsal corner only slightly produced beyond ventral corner	*Michotamia assamensis*

#### Genus Michotamia

Macquart

http://species-id.net/wiki/Michotamia

Michotamia Macquart, 1838, 1(2): 72. Type species: *Michotamia analis* Macquart, 1838, monotypic. Hull 1962, 2: 438 [genus description, species list]. Oldroyd 1975, 2: 130 [catalog]; 1980: 346 [catalog]. Daniels 1989: 333 [catalog]. Joseph and Parui 1998, 1: 169 [revision, Indian species]. Scarbrough and Hill 2000: 347 [Sri Lanka].
Allocotosia Schiner, 1866, 16: 845. Type species: *Asilus aurata* Fabricius, 1794; by original designation.
Allocotasia Wulp, 1872, 7: 249. *Lapsus calarni*.

##### Diagnosis.

Slender, medium [15-20 mm] flies with sparse, short setae on the thorax. *Head*: Face ventrally with unusually long, stout bristles; proboscis thick, robust with numerous ventral setae, the latter often clustered apically; postpedicel attenuate, as least 1.5 as long as scape and pedicel combined, and at least 1/2 as long as stylus; wide stripe of long, abundant setae present on lower 1/2 of frons; ocellarium with two short, thin setae. *Thorax*: Mesonotum with dorsocentral bristles either thin or absent; scutellar groove absent. *Wing*:Mostly or entirely dense microtrichose. *Leg*: Row of anteroventral bristles absent. *Abdomen*:Usually clavate. *Terminali*a:Aedeagus long, often thick, curved dorsally, with apex at or beyond cercus; sternite 8 in females with short V-shaped notch along apical margin apically, a short furrow or fissure, broad X-shaped or U-shaped apodeme internally (Scarbrough 2010; Scarbrough and Tomasovic 2010).

##### Remarks.

The long postpedicel is useful in diagnosing most species of *Michotamia*. A few undescribed species of *Ommatius* (Scarbrough, Dikow, & Tomasovic, in manuscript)and *Ommatius specious* Scarbrough & Hill, 2000 and *Ommatius sparsus* Scarbrough & Hill, 2000 have an unusually long postpedicel, i.e. ~1.5 times as long as the pedicel and scape combined. In addition, *Michotamia* has a low flattened mesonotum in which the maximum vertical height is less than 1/2 the length of the mesonotum, absence of stout anteroventral bristles on the hind femur, and a much longer, curved dorsad aedeagus distinguish the species. Further, a V-shaped apical notch medially which ends in a narrow fissure and a stout X- or U-shaped apodeme on the internal surface of sternite 8 distinguish females. In *Ommatius*, the postpedicel is usually is only as long as the pedicel and/or scape, rarely much longer; the mesonotum is strongly arched with the maximum vertical height being nearly 2/3 the length of the mesonotum; presence of stout anteroventral bristles on the hind femur; and a much shorter aedeagus, never curved dorsad and reaching the level of the cercus distinguish the species. Females lack a V-shaped apical notch medially that terminates in a fissure posteriorly and a stout X- or U-shaped apodeme on the internal surface of sternite 8.

#### Michotamia assamensis

Joseph and Parui, 1995

http://species-id.net/wiki/Michotamia_assamensis

Michotamia assamensis Joseph & Parui, 1995: 14. Type locality: India: Amsoi Forest (26°00'46.57"N, 92°32'46.14"E), Assam; Joseph and Parui 1998: 172.

##### New records.

**China: Yunnan:** 1 male, Xishuangbanna, Xiaomengyang (22°05'15.15"N, 100°53'57.92"E), 850m, 1957. IX.7, Shuyong Wang; 1 female, Xishuangbanna, Xiaomengyang, 850 m, 1957. X.11, Lingchao Zang. **Laos: Vientiane:** 1 male, Ban Van Eue, (17°57'48.72"N, 102°36'50.01"E) 1965.XI.30 ~ Native collector, Bishop Museum; 1 male, same data except 1966.II.15, native collector, malaise trap; 1 male, same data except 1966.III.30; 1male, Vientiane, Ci Sion, Vill. de Tha Ngone (18°07'56.86"N, 102°37'41.44"E), 1966.X.24-31, ~ Native collector.

##### Diagnosis.

Antennal pedicel brownish yellow, scape and postpedicel black; postpedicel 2.5 times as long as scape and pedicel combined. Fore and mid femora yellowish-orange, black extending from base to near apex dorsally and anteriorly; hind femur wider medially, 5 times longer than diameter, mostly black, basal 1/5 reddish-yellow or yellow; epandrium of male genitalia wide with parallel dorsal and ventral margins, apex subtruncate, slightly emarginated, dorsal corner only slightly beyond ventral corner.

##### Distribution.

China: Yunnan; India (Assam); Laos (Vientiane).

#### Michotamia aurata

(Fabricius, 1794)

http://species-id.net/wiki/Michotamia_aurata

Asilus aurata Fabricius, 1794: 387. Type locality: East India.
Lochites testaceus Bigot, 1878: (10)1, 425. Type locality: Myanmar (=Burma).
Michotamia aurata Oldroyd, 1975, 2, 130; Joseph and Parui (113), 36; 1998, 173.

##### New records.

**China: Hainan:** 2 males, 1934.VIII.31, Qi He; 1 female, Qiongzhong (19°02'00.13"N, 109°50'18.20"E), 400 m, 1960. VII. 14, Fushang Li.**Yunnan:** 2 females, Lancang (22°33'11.19"N, 99°55'55.56"E), 1000 m, 1957.VII. Lingchao Zang.

##### Diagnosis.

Antennal scape and pedicel yellow, postpedicel black and twice as long as scape and pedicel combined. Dorsocentral and scutellar bristles absent. All legs yellow, brownish yellow or reddish yellow. Wing with anterior basal half pale yellow and the rest infuscated, r-m well beyond middle of discal-cell.

##### Distribution.

China: Hainan, Yunnan, Taiwan; Bangladesh; India (Andaman Islands, Andhra Pradesh, Bihar, Karnataka, Kerala, Madhya Pradesh, Orissa, Pondicheryy, Tamil Nadu, Uttar Pradesh, and West Bengal); Indonesia (Maluku Islands, Sulawesi); Laos; Myanmar; Pakistan; Sri Lanka (Amparai, Anuradhapura, Colombo, Galle, Hambantota, Kandy, Matara, Mannar, Monarapala, Polonnaruwa, Trincomalee, Vavuniya); Thailand.

#### Michotamia subnigra nom. n.

http://species-id.net/wiki/Michotamia_subnigra

Michotamia nigra Scarbrough and Hill, 2000: 357. Type locality: Sri Lanka: Kan.: Kandy: Udawattakele Sancturay (7°17'55.96"N, 80°38'32.65"E). [preoccupied by *Michotamia nigra* (Meijere, 1911: 312 (Java).]

##### Note.

Scarbrough and Hill (2000) described *Michotamia nigra* from Sri Lanka. Unfortunately they failed to note that the binomen *Michotamia nigra* (Meijere, 1911) had been used earlier for a species from Java. We propose *Michotamia subnigra* as a replacement name for *Michotamia nigra* Scarbrough and Hill.

##### Distribution.

Sri Lanka.

#### Michotamia yunnanensis sp. n.

urn:lsid:zoobank.org:act:27E799F0-7FB8-40F3-B0C6-7A4EB465BCC6

http://species-id.net/wiki/Michotamia_yunnanensis

Figs 1 -7

##### Diagnosis.

Dorsal postocular bristles black and strong, middle and lower postocular bristles pale and thinner. Antenna black, postpedicel 3.5 times longer than scape and pedicel combined. Fore and mid femora yellow, dorsal surface of hind femur mostly blackish-brown, base narrowly yellow. Wing hyaline; crossvein r-m at apical 1/3 of discal cell.

##### Description.

Male. Body length 13 mm, wing length 10 mm.

**Figures 1–5.**
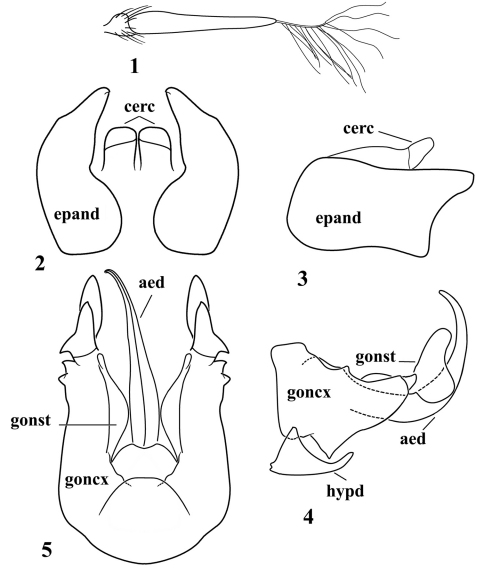
*Michotamia yunnanensis* sp. n. **1** antenna **2–3** epandrium & cercus (dorsal and lateral views) **4–5** hypandrium & gonocoxites (ventral and lateral views).

Head. Face brown, sparsely pale haired below antenna and with 2 vertical rows of 6 black bristles on lower 2/3, mystax with strong, yellow bristles; frons blackish-brown, several black bristles laterally; vertex blackish-brown; occiput with pale hairs, its lower portion with long pale hairs; dorsal postocular bristles black, middle and lower postocular bristles pale. Antenna (Fig. 1) black, wide apex of scape and pedicel reddish, postpedicel black, 3.5 times longer than scape and pedicel combined; stylus brown, less than 1/2 as long as postpedicel. Proboscis black, pale hairs basally and apically; palpus black, with black hairs and bristles.

**Figures 6–7.**
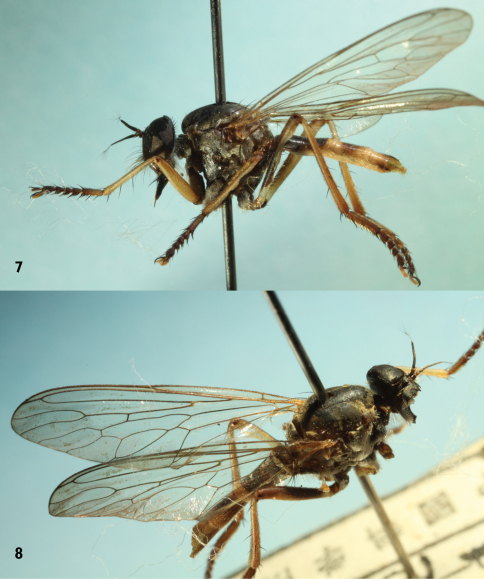
*Michotamia yunnanensis* sp. n. **7** Habitus, lateral **8** dorsal views.

Thorax. Black with white pubescence. Mesonotum black with golden yellow pubescence laterally; 2 dc, 2 npl, 1 spal and 1 pal. Scutellum black with pale hairs and 2 weak marginal scutellar setae. Pleuron wholly black, with dense pale white pubescence. Katatergite with a row of 7 brown bristles. Wing (Figs 6–7) hyaline, tinged grayish apically; veins basally yellowish and apically brown to blackish; crossvein r-m at apical 1/3 of discal cell. Anal cell closed with short stalk. Halter yellow.

Legs (Figs 6–7). Largely yellow; coxae black, with dense pale pubescence and strong pale bristles. Fore and mid femora yellow with black tip, hind femur mostly yellow, blackish anterodorsally. Tarsi reddish-brown except basal half of tarsomere 1 brownish-yellow. Legs with most hairs and bristles black. Fore tibia with 1 av, 2 ad and 2 pd bristles, mid tibia with 2 ad; hind tibia with 1 av, 2 ad and 2 pd bristles. Claws black.

Abdomen (Figs 6–7) with long pale hairs laterally and shorter brown hairs dorsally. Abdominal tergite 1 black, tergite 2 black with yellow band posteriorly, tergites 3–4 blackish at middle, remaining tergites black; abdominal sternites 1–3 yellow, sternite 4 brownish. Abdominal segments 5–7 black. Male genitalia yellow (Figs 2–5). Epandrium wide basally, apex angular and with shallow emargination, dorsal corner produced well beyond ventral corner. Hypandrium somewhat triangular.

Female. Unknown.

##### Type material.

Holotype ♂, Yunnan: Xishuangbanna, Menghun (21°50'31.37"N, 100°23'08.00"E), 750 m, 1958.VI.1, Chunpei Hong.

##### Etymology.

The species name *yunnanensis* refers to the Province of Yunnan.

##### Remarks.

*Michotamia yunnanensis* sp. n.is distinguished from *Michotamia assamensis* by the hyaline wings (Figs 6-7), color of the femora as described in the key, and the combined characters of the terminalia, especially the shape of the epandrium (Figs 2-5). In *Michotamia assamensis*, the fore and mid femora are yellowish-orange ventrally and posteriorly, black dorsally and anteriorly from base to near apex, the hind femur is mostly black with the narrow base yellowish-orange, and the wing is dark brownish-yellow and basal 1/3 of the anal lobe is hyaline (Joseph and Parui 1995, 1998).

##### Distribution.

China: Yunnan.

## Supplementary Material

XML Treatment for Michotamia

XML Treatment for Michotamia assamensis

XML Treatment for Michotamia aurata

XML Treatment for Michotamia subnigra

XML Treatment for Michotamia yunnanensis

## References

[B1] Bigot JMF (1878) Diptères nouveaux ou peu connus. 10. partie. XV. Tribu des Asilidae. Annales de la Societe entomologique de France 5(7): 73–74

[B2] Daniels G (1989) Family Asilidae. In: Evenhuis NL (Ed). Catalog of the Diptera of Australasian and Oceanian Regions.Bishop Museum Press, Honolulu, 86: 326-374

[B3] Geller-Grimm F (2003/2004) Catalogue of the species of Asilidae. Available from http://www.geller-grimm.de/catalog/species.htm [Accessed 29 March 2012]

[B4] Hua LZ (1985) A list of robber flies from China (Diptera: Asilidae). Institute of Entomology, Zhongshan Universty, Guangzhou, 1–17

[B5] Hull FM (1962) Robber flies of the world. The genera of the family Asilidae. United States National Museum Bulletin, Washington DC, 224(1/2), 907 pp.

[B6] Fabricius JC (1794) Entomologia systematica emendata et aucta. Secundum classes, ordines, genera, species, IV. Hafniae, Copenhagen, 376–390

[B7] Joseph ANT Parui P (1983) A review of the Asilidae from the Oriental Region. Oriental Insects 17: 269-393 doi: 10.1080/00305316.1983.10433697

[B8] Joseph ANT Parui P (1984) Studies on the Asilidae (Diptera) collections made by Dr. Ghorpade. Records of the Zoological Survey of India 66: 1-140

[B9] Joseph ANT Parui P (1987a) On some Asilidae (Diptera) from India. Bulletin of the Zoological Survey of India 8(1/3): 89–109

[B10] Joseph ANT Parui P (1987b) On some Asilidae (Diptera) from India present in the Smithsonian Institution. Oriental Insects 21: 147-162

[B11] Joseph ANT Parui P (1995) On Asilidae (Diptera) from India & adjacent countries present in the California Academy of Sciences. The Wasmann Journal of Biology 50(1/2): 1–38 [cited as 1994 in Joseph and Parui's paper in 1998]

[B12] Joseph ANT Parui P (1998) The fauna of India and the adjacent countries. Diptera (Asilidae) (part 1). Zoological survey of India, Calcutta, 278 pp.

[B13] Macquart PJM (1838) Diptères exotiques nouveaux ou peu connus. Mémoires de la Société royale des Sciences1838 de l`Agriculture et des Artes 1(2): 5–207

[B14] Meijere JCH de (1911) Studien über südostasiatische Dipteren VI. Tijdschrift voor entomologie 54: 258–432, 300–322

[B15] Oldroyd H (1975) Family Asilidae. In: Delfinado E Hardy MD (Eds). A catalog of the Diptera of Oriental region, II.University Press of Hawaii: 99-156

[B16] Scarbrough AG (2010) An overview of the Afrotropical Ommatiinae (Diptera: Asilidae) with a key to genera. Zootaxa 2540: 1-47

[B17] Scarbrough AG Tomasovic C (2010)*Ommatomyia*,a new Ommatiinae genusfromVietnam (Diptera: Asilidae: Ommatiinae). Zootaxa 2366: 46-54

[B18] Scarbrough AG Hill HN (2000) Ommatiine robber flies (Diptera: Asilidae) from Sri Lanka. Oriental Insects 34: 341-407 doi: 10.1080/00305316.2000.10417277

[B19] Schiner JR (1866) Nachtrag zu Schiner`s Vortrag über die Asiliden Wiedemann`s. Verhandlungen der zoologisch-botanischen Gesellschaft in Wien 16: 845-848

[B20] Stuckenberg BR (1999) Antennal evolution in the Brachycera, with a reassessment of terminology relating to the flagellum. Studia Dipterologica 6 (1): 33-48

[B21] Tomasovic G Grootaert P (2003) New Asilidae (Diptera) from Thailand: contribution 1. Bulletin de la Société royale belge d'Entomologie 139(7/12): 252–258

[B22] Tomasovic G Grootaert P (2008) Four new species of robber flies (Diptera: Asilidae) from the Oriental Region. Bulletin de la Société royale belge d'Entomologie 144: 71-78

[B23] Wulp FM van der (1872) Bijdrage to de Kennis der Asiliden van den Osst-Indischen Archipeligo. Tijdschrift voor Entomologi 7: 139-279

